# Scabies Outbreak Investigation and Risk Factors in East Badewacho District, Southern Ethiopia: Unmatched Case Control Study

**DOI:** 10.1155/2018/7276938

**Published:** 2018-06-26

**Authors:** Jarso Sara, Yusuf Haji, Achamyelesh Gebretsadik

**Affiliations:** ^1^Affiliate of School of Public and Environmental Health, College of Medicine and Health Sciences Hawassa University, Ethiopia; ^2^School of Public and Environmental Health, College of Medicine and Health Sciences, Hawassa University, P.O. Box 1560, Hawassa, Ethiopia

## Abstract

**Introduction:**

Scabies is one of the common public health problem but neglected parasitic diseases caused by* Sarcoptes scabiei *var.

**Methods:**

A community-based unmatched case control (1 : 2 ratios) study was conducted in East Badewacho District, using collected scabies line listed data and face-to-face interview to assess risk factors during October 23–30, 2016. The data were collected using structured questionnaire, and then the data were coded, entered, cleaned, and analyzed using SPSS statistical software, whereas, line listed data was entered into Microsoft excel for descriptive analyses. Odds ratios (OR) and 95% confidence interval (CI) were computed to determine associated factors.

**Results:**

A total of 4,532 scabies cases line listed with overall attack rate of 110/1,000 population. The mean age was 12 years, and most affected age group was 5–14 years. Independent risk factors found to be statistically associated with scabies infestation were age less than 15 years (AOR = 2.62, 95% CI: 1.31–5.22), family size greater than 5 members (AOR = 2.63, 95% CI: 1.10–6.27), bed sharing with scabies cases (AOR = 12.47, 95% CI: 3.05–50.94), and home being affected by flooding (AOR = 22.32, 95% CI: 8.46–58.90).

**Conclusion:**

Outbreak of scabies occurred in East Badewacho District. Age less than 15 years, family size greater than five members, sleeping with others, and home being affected by flooding are the risk factors. Providing risk factors related health education on prevention and controls especially, at community level and schools, is recommended.

## 1. Introduction

Scabies is one of the common but neglected parasitic diseases and is major public health problem globally and in resource-scarce countries in particular. Global scabies prevalence was about 204 million cases with 0.21% of total disability adjusted life years lost, and, in resource-poor tropical settings, the sheer burden of scabies infestation and their complications impose a major cost on healthcare systems [1, 2].

Scabies affects all age groups and both sexes but the most vulnerable age groups are young children and the elderly in resource-poor communities who are especially susceptible to scabies as well as to the secondary complications of infestation. The highest rates occur in countries with hot, tropical climates, where infestation is endemic, especially in communities where overcrowding and poverty coexist [1, 3].

The scabies mites usually spread by prolonged direct skin-to-skin contact with a person who has scabies. It can also spread easily to sexual partners and household members. Sometimes scabies can spread indirectly by sharing clothes, towels, or bedding used by infested individuals. A tiny scabies mite burrows into the epidermis of the skin where it lives and lays its eggs. The most common symptoms of scabies are severe itching especially at night and papular skin rash that may affect much of the body or be limited to common sites like interdigital space, flexor of the wrist, elbow, armpit, penis, nipple, and buttocks which usually begin 3–6 weeks after primary infestation [1, 4].

An outbreak of scabies could happen when cases are left untreated, and delayed diagnosis is linked with secondary bacterial infection which may lead to cellulitis, folliculitis, boils, impetigo, or lymphangitis and may also exacerbate other preexisting dermatoses such as eczema and psoriasis [5]. These secondary bacterial infections were mostly caused by group A streptococci and* Staphylococcus aureus*, which leads to nephritis, rheumatic fever, glomerulonephritis, chronic renal and rheumatic heart diseases, and sepsis especially in developing countries that causes for many deaths [6]. And evidence of renal damage is as high as 10% of children with infected scabies in resource-poor settings [1].

It is reported that overcrowded living conditions, sleeping together, sharing of clothes, sharing of towels, poor hygiene practices, malnutrition, and travel to scabies outbreak areas are common risk factors for scabies [1, 7, 8].

In Ethiopia, scabies is also common especially during natural or manmade disasters such as flooding, drought, civil war and conflict, poor water supply and sanitation, and overcrowding living condition. For example, according to public health emergency measures surveillance report scabies is becoming beyond sporadic clinical cases but is turn to be a public health concern and affecting wider geographic areas and population groups especially in drought affected nutrition hotspot woredas [9]. Previous study reported that the prevalence of scabies in tropical counties was high; for example, in Fiji the prevalence of scabies in school children was 18.5% [10]. A study in Northern Ethiopia, Gonder town, among “Yekolo Temari”, revealed 22.5% scabies prevalence; however, another study conducted in southern Ethiopia revealed a prevalence of 5.5% among school children [11, 12]. Currently, Ethiopia is experiencing scabies outbreak in drought affected areas where there is shortage of safe water for drinking and poor personal hygiene as a result of direct impact of the drought caused by El Niño [13]. However, there is lack of studies regarding outbreak investigation and risk factors in the current study areas. Therefore, this study was aimed at investigating the scabies suspected outbreak and its risk factors in East Badewacho District, Southern Ethiopia.

## 2. Methods and Materials

### 2.1. Study Area and Population

**Figure 1:**
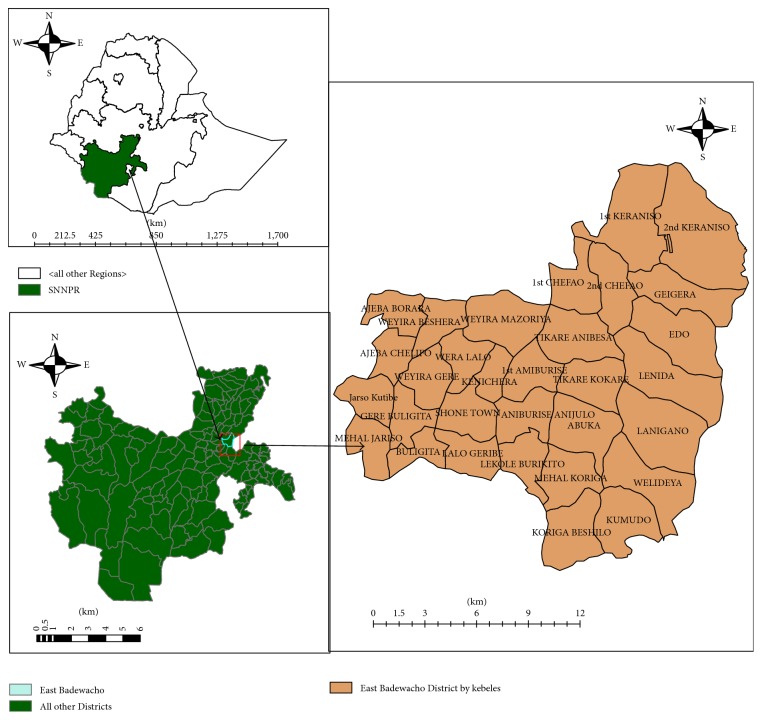
Administrative map of study kebeles, East Badewacho District, Southern Ethiopia, 2016.

The study was conducted in East Badewacho, one of the 11 districts of Hadiya Zone, Southern Nations Nationalities and Peoples (SNNPR) State of Ethiopia. Administratively, the district has 39 kebeles/subdistricts (1 urban versus 38 rural). As projected from 2007 Ethiopian Population Census, the 2016/17 population of the district is estimated to be 171,578 (85, 275 males, 86,303 women). Shone town, the district capital, is located at 90 km from Hosaina, the Zonal capital, and 115 km from Hawassa city, the Regional capital in the southwest, and 340 km from Addis Ababa, capital city of Ethiopia. The kebeles/subdistricts at which investigation conducted were 1st Chefa, 2nd Chefa, 1st Kerranso, 2nd Kerranso, and Gegara located nearly 15, 17, 23, 27, and 30 km, respectively, away from the Shone town and selected purposely (Figure 1). These kebeles/subdistricts were affected by flooding disaster occurred in 2016 El Niño in the region [13].

Majority (80.6%) of district populations live in the rural while the remaining 19.4% were urban dwellers. The district has an area of 308.85 square kilometers with average population density of 555 people per square kilometers.

Currently, the district has 1 district hospital, 7 health centers, 41 health posts, and 21 private clinics which accounts for 98% of potential health services coverage. The overall water supply coverage of the district was 38%.

### 2.2. Study Design and Period

We conducted community-based unmatched case control (1 : 2 ratio) study from October 23–30/2016 to identify potential risk factors and ways of transmission. Line listed data analysis was performed.

### 2.3. Data Collection Methods and Tools

We used a structured questionnaire, which is adapted from different literatures, to collect information including sociodemographic characteristics, clinical features and management of the cases, and the possible risk factors. The data were collected through face-to-face interview with individual participants, or their families in case of children. Two unmatched controls were selected per each case. Line listing of cases was collected from health facilities and schools for further analysis. Data were collected by two trained diploma nurses.

### 2.4. Inclusion and Exclusion Criteria

#### 2.4.1. Inclusion Criteria

*Cases*. Any resident of the kebeles, East Badewacho District, with sign and symptoms (specifically itching and rash) of scabies was selected for investigation and agreed to participate in the study during investigation period.

Diagnosis of a scabies infestation usually is clinical, made based upon presence of the typical rash and symptoms of unrelenting and worsening itch, particularly at night [9].

*Controls*. Any resident of community of kebeles without any signs and symptom of scabies was selected during the investigation period and agreed to participate in the study.

#### 2.4.2. Exclusion Criteria

*Cases*. Those who refused to participate or none residents of the selected kebeles were excluded.

*Controls*. Those who refused to participate as well asfamily members from the same household were excluded from the study (if there are two or more persons in a single household, only one person randomly selected).

### 2.5. Data Analysis Procedures and Quality Control

Line listed data were entered and cleaned using Microsoft Office Excel 2007 for descriptive analysis, SPSS version 20 statistical software was used for risk factor identification and analysis. All line listed and interviewed data were checked for completeness before entry, cleaning, and analysis made.

Arc map was also used for mapping cases and the administrative area of the study. Results were presented using descriptive tables, charts, and choropleth map. Attack rate, *p*-value, and crude and adjusted ORs with 95% CI were used in deciding the strength and statistical significance of associations.

### 2.6. Study Variables

#### 2.6.1. Dependent Variable

Scabies infestation was a dependent variable.

#### 2.6.2. Independent Variables

Sociodemographic (age, sex, occupation, marital status, religion, and family size), travel history, contact history, adequacy of water for personal hygiene, and overcrowding condition were independent variables.

### 2.7. Ethical Consideration

**Figure 2:**
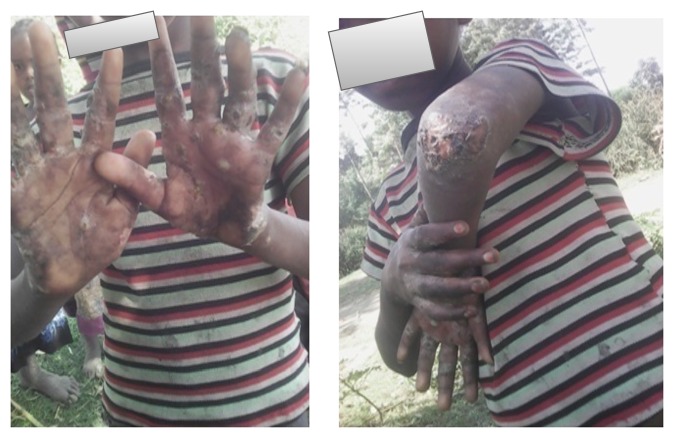
An 8-year-old girl of Gegara primary school student with secondary infection, Gegara kebele, East Badewacho District, Southern Ethiopia, 2016.

**Figure 3:**
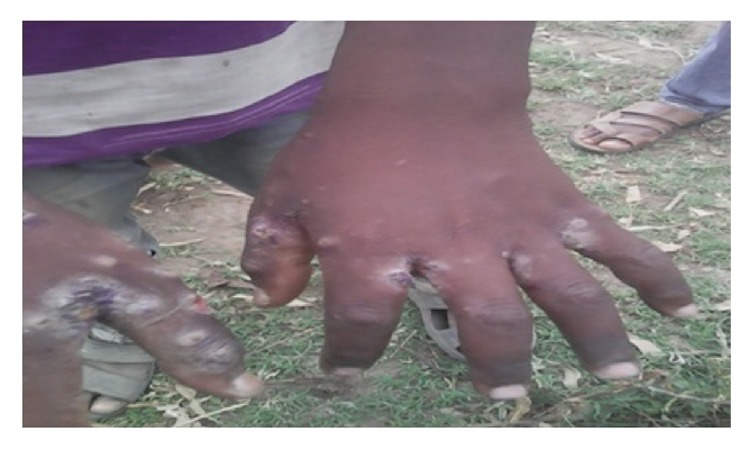
A 10-year-old boy's hands at 1st Kerenso primary school with secondary infection, 1st keranso kebele, East Badewacho District, Southern Ethiopia, 2016.

Letter of permission was obtained from SNNPR State Health Bureau, Public Health Emergency Management (PHEM) core process and other concerned organizations. Informed verbal consent was also obtained from all the study participants, or their parents in case of children. For the sake of confidentiality the names of participants were not recorded on the questionnaire. Regarding Figures 2, 3, and 7, informed consent was also obtained again from concerned participants orally and their names were not written on the figures.

### 2.8. Case Definition

#### 2.8.1. Suspected Case

A person with signs and symptoms consistent with scabies was suspected. The characteristic symptoms of a scabies infection include superficial burrows, intense pruritus (itching) especially at night, a generalized rash, and secondary infection on the head, face, neck, armpit, elbow, wrist, palms, buttocks, and soles [4, 9].

#### 2.8.2. Confirmed Case

A person who has a skin scraping in which mites, mite eggs, or mite feces have been identified by a trained healthcare professional was considered a confirmed case [4, 9].

#### 2.8.3. Contact

Contact is defined as a person without signs and symptoms consistent with scabies who has had direct contact (particularly prolonged, direct skin-to-skin contact) with a suspected or confirmed case in the two months preceding the onset of scabies signs and symptoms in the case [4, 9].

## 3. Result

### 3.1. Description of Line Lists

Rumors of scabies cases were reported from two primary schools (Gegara and 2nd keranso), East Badewacho District, on 17 October, 2016. From October 23 to 30, 2016, we identified a total of 4,532 suspected scabies cases line listed from 9 kebeles of the district with a prevalence of 11% (4532/41287). The overall attack rate of nine affected kebeles was 110 cases/1,000 populations, with no scabies related death (CFR = 0).

Out of 4,532 total suspected scabies cases, 2633 (58%) of them were males while 1,899 (42%) were females. The mean age was 12 years, ranging from 8 months to 70 years. Children of 5–14 years of age were the most affected age group with an attack rate of 263/1000 population followed by 15 years and above age groups which accounts for 41/1000 population (Table 1).

Most affected populations were children in the primary schools and most of them had shown sign of secondary infection attributable to scabies. For example, Figures 2 and 3 indicate cases with secondary infection captured during investigation.

**Figure 4:**
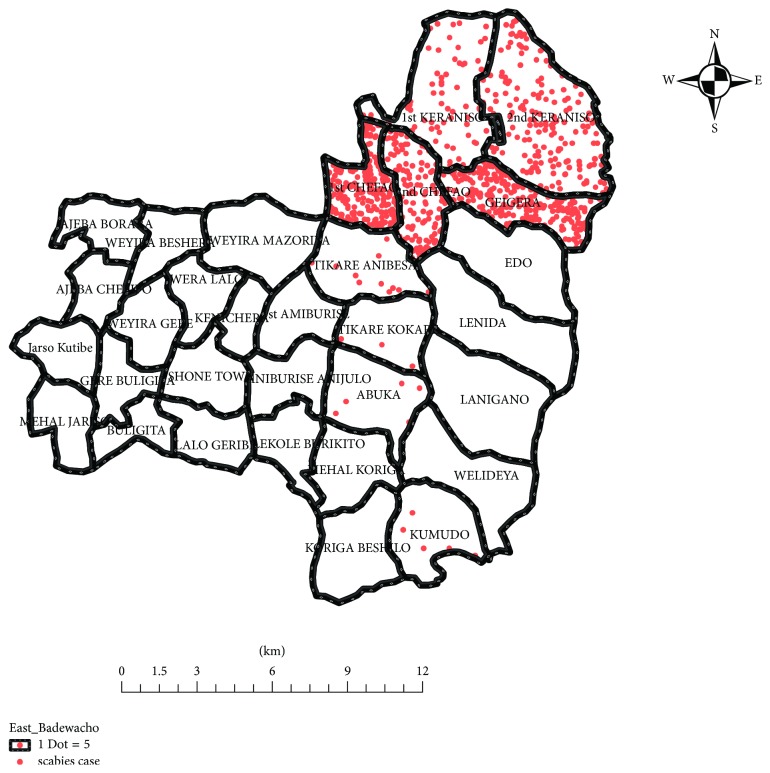
Spot map of scabies cases by kebeles, East Badewacho District, Southern Ethiopia, 2016.

During investigation period, 29% of the cases were reported from Gegara kebele followed by 1st Chefa (25%) and 2nd Keranso (20.5%) kebeles, whereas small numbers of cases were reported from Tikare Kokare (0.4%) and Abuka (0.6%) kebeles.  Figure 4 shows the spot map of cases by kebeles.

Age-specific attack rate (ASAR) was highest among the age group of 5−14 (85/1000 population) with 227/1000 population in 1st Chefa followed by Gegara (220/1000 population) kebele (Table 2).

**Figure 5:**
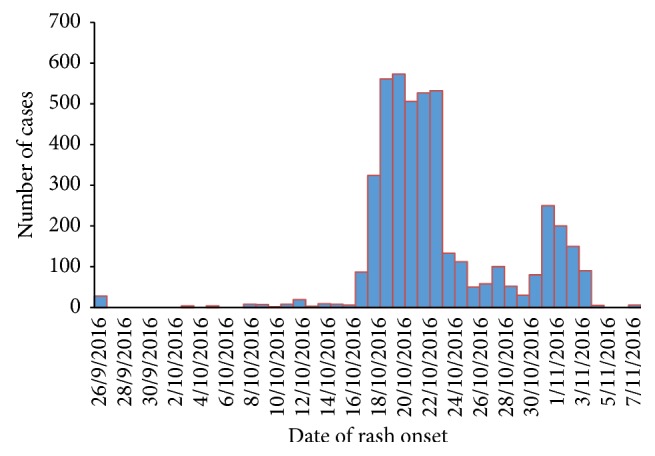
Epidemic curve of scabies outbreak by date of onset, East Badewacho District, Southern Ethiopia, 2016.

On October 19, 2016, district heath office notified the situation to Hadiya Zone Health department. Then, Zonal Health Department notified the situation to Regional Health Bureau (RHB) on October 20, 2016, and investigation team was deployed to assess the situation (Figure 5).

### 3.2. Case Control Analysis

A total of 165 (55 cases and 110 controls) participants were randomly selected from the community to identify the risk factors for scabies outbreak in affected 9 kebeles with case to control ratio of 1 : 2. Almost all cases had a history of rash and itching, and 33 (60%) of them had sign of secondary infection. Among the total 55 interviewed cases, 33 (60%) of them were males and 22 (40%) were females; and of 110 controls, 62 (56%) were males and 48 (44%) of them females. The mean age of study subjects was 12.62 (6 months–65 years) years of age among cases, while the mean age for controls was 20.8 years (3 months to 50 years), *p*. value < 0.01 (Table 3).

#### 3.2.1. Presence of Clinical Features of Scabies

**Figure 6:**
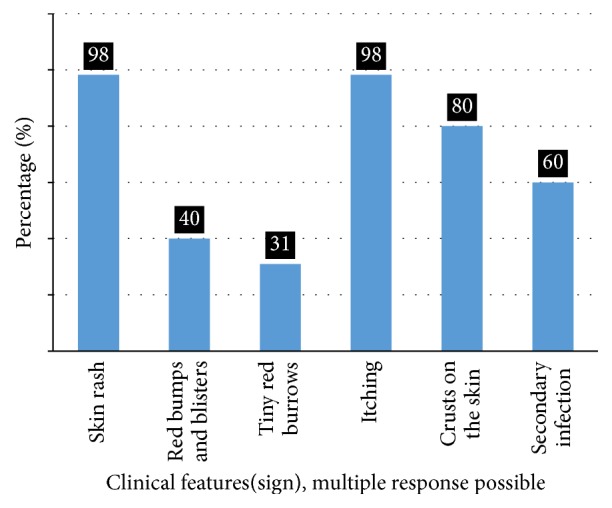
Percentage of cases with clinical features of scabies, East Badewacho District, Southern Ethiopia, 2016.

**Figure 7:**
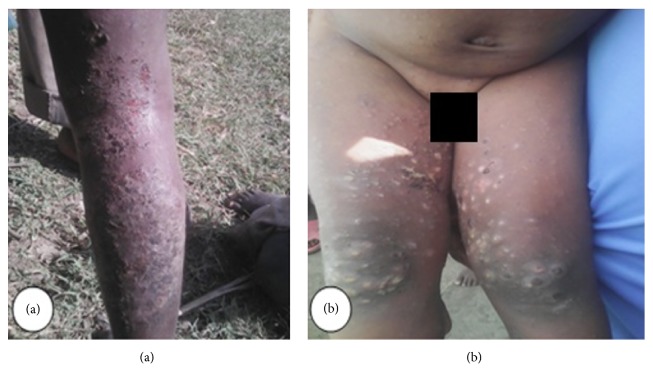
Posterior leg (a) and intergluteal (b) scabies rash on the body, East Badewacho District, Southern Ethiopia, 2016.

Figure 6 shows clinical features of scabies diagnosed subjects (cases). Accordingly, of 55 cases, 54 (98%) of them had both itching and scabies related skin rash, followed by crusts on the skin that was not yet ascertained as that of scabies crusts 44 (80%) and secondary bacterial infection 33 (60%).

Of the total cases, 35 (64%) of them did not visit health facility to get treatment for infestation. Thirteen percent of cases and 14% of controls had travel history within past 2 months prior to the onset of symptoms. Fifty-two (94%) of cases responded that they had contact history with active case of scabies. However, 84 (76%) of controls reported that they had no history of contact with scabies cases. Among the cases those who had a contact history, 32 (58%) of them had history of sleeping together, 30 (54%) playing together, and 18 (33%) sharing clothes as types of contacts.

Regarding site of the rash on the body, 41 (75%) of cases had it on buttocks, 39 (71%) had it on interdigital space, 35 (64%) of cases had rash on the flexor wrist surface, and the rest are stated in table (Table 4).

#### 3.2.2. Factors Associated with Scabies Outbreak

Concerning risk factors, variables such as sex, age, educational status, religious, marital status, family size, travelling history to scabies epidemic area within the last 2 months, sleeping with scabies cases, water source for daily bases, and home being affected by flooding in last disaster were entered into binary logistic regression model.

In bivariate analysis, age group, family size > 5 members, sleeping with person infested with scabies cases, water source for daily bases, and home being affected by flooding were significantly associated with scabies infestation (Table 5).

Age in years, family size, sharing beds with scabies cases, source of water for daily bases, and home being affected by flood were entered into multivariate logistic regression model to control for confounding factor.

After adjusting for possible confounding factors the result of multiple logistic regression analysis showed that age group less than fifteen years, family size > greater 5 members, sleeping with scabies cases, and home being affected with flood were found to be the final independent variables significantly associated with scabies infestation. Accordingly, those persons aged less than 15 years were 2.6 times more likely to develop scabies with [AOR (95% CI) = 2.62 (1.31–5.22)] compared with age >= 15 years of age. The odd of developing scabies infestation was 2.6 among family members with size >= 5 persons compared to those whose family size <= 5 members with [AOR (95%) = 2.63 (1.10–6.27)]. There is also strong association between the home being affected by flooding and scabies infestation. Thus, the odd of acquiring scabies was about 22 times among households affected by flooding than their counterparts [AOR (95% CI) = 22.32 (8.46–58.90)].

## 4. Discussion

We identified a total of 4,532 suspected scabies cases line lists from 9 kebeles, and 165 individuals were randomly selected from the community as case-controls (55 cases and 110 controls) from 5 purposely selected kebeles/subdistricts during investigation period. The overall prevalence rate was 11% with school-aged children being affected more (26%). This result is lower to the findings of studies conducted in Northern Ethiopia among “Yekolo temeri” which is reported to be 22.5% and study among school children in Fiji where the overall prevalence of scabies infection was 23.6% [10, 11]. The relative lower prevalence observed in our study could be due to the fact the study population mostly institutionalized, for example, “yekolo temeri”, in Ethiopia was mostly living in single institution (church), while this study conducted among the general rural community, and, as to Fiji study, the climatic variation might have existed as Fiji is tropical climate and ours is subtropical area.

However, the finding of this study is higher than other study conducted in southern Ethiopia reporting 5.5% scabies prevalence among school children [12]. This is because, our study conducted in drought hit areas, El Nino was the case in the study area [13].

In this study 75% of cases were found in the age group of 5 to 14 years, and age groups less than 15 years are at risk of acquiring scabies compared to age greater than 15 years. Our findings are similar to studies conducted in Fiji and Cameroon where the school-aged children commonly affected [10, 14]. Children in primary school were most affected populations, and most of them had sign of secondary infection attributable to scabies. This might be due to the fact that younger children, particularly, those at school are at high risk of scabies infestations as the school environments may increase the susceptibility of cross-infestation and increase contacts which can be passed to family members and other.

Regarding the sites of rash, interdigital spaces (71%), flexor wrists (64%), and buttocks (75%) were the main sites in the current study. This is nearly similar to the study conducted at boarding schools in Cameroon with the interdigital spaces and flexor wrists were the common sites affected by scabies [14]. This might be true as these parts of the body might be softer than the other body parties which is favorable for mites [1].

Concerning the risk factors for scabies, there is statistically significant associations between family sizes and scabies infestation that the odds of acquiring scabies are higher in those having more than five family members. This finding is consistent with study done in Solomon Islands indicating households with six to ten persons per household were 1.4 times more likely to acquire scabies compared to those households having less than 5 family members [15]. In addition, this result is also supported by other similar study conducted in west of Iran which revealed that scabies had been directly associated with family size [16]. This might be due to overcrowding among larger families compared to the smaller ones, which increases sharing of cloths, beds, etc. It is well known that scabies can spread easily under crowded conditions where close body and skin contact is common [7].

Another factor showing strong association is sleeping with cases of scabies. Hence, those who had reported to have slept with scabies infested individuals were twice more likely to have developing scabies infestations than their counterparts. This result is in line with systematic reviews done on scabies in developing countries showing that having skin contact in the past 2 months with a person infested with scabies and sleeping with others were risk factors for scabies [17]. Moreover, study conducted among solders in Pakistan shows sharing of beds among male soldiers was one of the risk factors for scabies [8]. This is supported by a body of science that mites of scabies can be frequently transmitted by skin-to-skin contacts, as well as through infected closes and bedding [1, 4, 5].

Moreover, strong association was seen between homes being affected by flooding and scabies infestations in the current study. Compared to controls, the odds of households being affected by flooding were 22 times among that of cases. This might be a result of the displacements, overcrowding, and impairment in personal hygiene and may increase susceptibility to different skin problems like scabies infestation.

Finally, we recommend the following:

- Strong and continuous active case search should be strengthened at all levels.
- Providing risk factors related health education on prevention and control especially is recommended, at schools and community level.
- Scabies mass drug treatment should be initiated as soon as possible in kebeles with prevalence ≥ 15%.
- As long as each scabies outbreak is unique and requires an individualized approach, we recommend maintaining social mobilizing at health facilities, schools, and any public gathering areas to alleviate the spread of scabies.

Our study is not free from limitations. First of all, as the study conducted is based on only a clinical signs and symptoms while lacking laboratory confirmation, ascertainment of cases could be a problem. Second, due to small sample size some confidence intervals are wider, for assessing risk factors for scabies. Another limitation of our study is ascertainment of clinical presentations of cases of scabies; for example, secondary bacterial infections, crusts, and presence of burrows are difficult as we did not employ any laboratory or microscopic test. Furthermore, as we employed case control study, the role of recall bias could not be ruled out.

## 5. Conclusions

In conclusion, it is confirmed that scabies outbreak occurred in East Badewacho District, Southern Ethiopia. Age less than 15 years, family size > 5 members, contact history with scabies cases, sleeping with others, and home being affected by flooding are the independent risk factors associated with scabies in the district.

## Figures and Tables

**Table 1:** Scabies attack rate by age-group of affected kebeles, East Badewacho District, Hadiya Zone, SNNP region, Ethiopia, October 23–30, 2016.

Age group	Age group population	Number of cases	Attack rate per 1,000
0–4	6,445	137	21
5–14	13,319	3,509	263
15+	21,523	886	41
*Total*	*41,287*	*4,532*	*110*

**Table 2:** Distribution of scabies cases by affected kebeles, East Badewacho District, Southern Ethiopia 2016.

Kebeles	Total population	0–4		5–14		≥15	
		cases	ASAR/1000	cases	ASAR/1000	cases	ASAR/1000
1st Chefa	3,730	42	11	845	227	265	71
1st Keranso	6,167	0	0	325	53	17	3
2nd Chefa	4,944	32	6	527	107	101	20
2nd Keranso	6,502	8	1	872	134	51	8
Abuka	5,487	0	0	18	3	9	2
Gegara	3,833	70	18	842	220	409	107
kumudo	2,465	0	0	10	4	13	5
TikareAnbasa	4,225	0	0	52	12	6	1
Tikarekokare	3,934	0	0	11	3	7	2
*Total*	*41,287*	*152*	*4*	*3502*	*85*	*878*	*21*

**Table 3:** Sociodemographic characteristics of the cases and controls, East Badewacho District, Southern Ethiopia, 2016.

Variables	Case, *n* (%)	Control, *n* (%)	Total, *n* (%)	*p*. Value
*Sex *				0.65
Male	33 (60)	62 (56)	95 (58)	
Female	22 (40)	48 (44)	70 (42)	
Age in years				<0.01
0–4	11 (20)	8 (7)	19 (12)	
5–14	24 (44)	31 (28)	55 (33)	
15–44	19 (35)	64 (58)	83 (50)	
45+	1 (2)	7 (6)	8 (5)	
Religion				0.39
Protestant	12 (22)	18 (16)	30 (18)	
Muslim	43 (78)	92 (84)	135 (82)	
Occupation				0.78
Student	24 (44)	40 (36)	64 (39)	
Unemployed	1 (2)	2 (2)	3 (2)	
Merchant	1 (2)	4 (4)	5 (3)	
Farmer	29 (53)	64 (58)	93 (56)	
Educational Status				0.74
No formal education	21 (38)	45 (41)	66 (40)	
Formal education	34 (62)	65 (59)	99 (60)	
Marital status				0.82
Single	24 (44)	50 (45)	74 (45)	
Married	31 (56)	60 (55)	91 (55)	
Family members				0.01
>5 persons	47 (85)	72 (65)	119 (72)	
≤5 persons	8 (15)	38 (35)	46 (28)	

**Table 4:** Site of the rash on the body of investigated cases, East Badewacho District, Southern Ethiopia, 2016.

Site of Rash	Number of cases (*n* = 55)	Percentage (%)
Flexor wrist surface	35	64
Inter digital spaces	39	71
Abdomen	30	55
Inter gluteal	33	60
Buttocks	41	75
Elbow	25	45

*Note*. Multiple responses possible.

**Table 5:** Bivariate and multivariate analysis of Scabies outbreak, East Badewacho District, Southern Ethiopia, 2016.

Variables/Risk factors	Case, *n* (%)	Control, *n* (%)	COR (95% CI)	AOR (95% CI)	Adjusted *p*-values
Sex					
Male	33 (60)	62 (56)	1		
Female	22 (40)	48 (44)	1.16 (0.601–2.24)		
Age in years					
<15	19 (35)	67 (61)	2.95 (1.503–5.798)	2.624 (1.31–5.22)	0.006
≥15	36 (65)	43 (39)	1	1	
Educational status					
Formal education	21 (38)	45 (41)	1		
No formal education	34 (62)	65 (59)	0.892 (0.459–1.732)		
Religious					
Muslim	43 (78)	92 (84)	1		
Protestant	12 (22)	18 (16)	1.426 (0.63–3.22)		
Marital status					
Single	24 (44)	50 (45)	1		
Married	31 (56)	60 (55)	1.098 (0.525–2.296)		
Family Size					
≤5	8 (15)	38 (35)	**1**		0.028
>5	47 (85)	72 (65)	4.10 (1.33–7.22)	2.63 (1.10–6.27)	
Travel history to scabies epidemic area within the last 2 months					
No	48 (87)	94 (85)	1		
Yes	7 (13)	16 (15)	0.857 (0.330–224)		
Sleeping with scabies cases					
Yes	32 (62)	4 (17)	7.6 (2.25–25.59)	12.4 (3.05–50.9)	<0.0001
No	20 (38)	19 (83)	1	1	
Source of water for daily bases					
Pipe Water	28 (51)	84 (76)	1	1	
Spring	4 (7)	2 (2)	3 (1.13–7.95)	5.57 (0.57–53.75)	0.137
Pond	10 (18)	10 (9)	6 (1.04–34.54)	2.36 (0.68–8.11)	0.171
River	13 (24)	14 (13)	2.786 (1.170–6.63)	1.63 (0.57–4.69)	0.358
Home affected by flooding					
Yes	49 (89)	82 (75)	23.9 (9.24–61.84)	22.32 (8.46–58.90)	<0.0001
No	6 (11)	28 (25)	1	1	

## Data Availability

The data supporting the summarization of the current article is included in the main article (as additional file).
